# Antimicrobial susceptibility, serotype distribution, virulence profile and molecular typing of piliated clinical isolates of pneumococci from east coast, Peninsular Malaysia

**DOI:** 10.1038/s41598-021-87428-z

**Published:** 2021-04-15

**Authors:** Nurul Diana Dzaraly, Mohd Nasir Mohd Desa, AbdulRahman Muthanna, Siti Norbaya Masri, Niazlin Mohd Taib, Zarizal Suhaili, Nurshahira Sulaiman, Nurul Hana Zainal Baharin, Cheah Yun Shuan, Zarina Ariffin, Nor Iza A. Rahman, Farahiyah Mohd Rani, Navindra Kumari Palanisamy, Tuan Suhaila Tuan Soh, Fatimah Haslina Abdullah

**Affiliations:** 1 Department of Biomedical Sciences, Faculty of Medicine and Health Sciences, Universiti Putra Malaysia, UPM Serdang, Selangor Malaysia; 2 Department of Medical Microbiology, Faculty of Medicine and Health Sciences, Universiti Putra Malaysia, UPM Serdang, Selangor Malaysia; 3 School of Animal Science, Aquatic Science and Environment, Faculty of Bioresources and Food Industry, Universiti Sultan Zainal Abidin, Besut Campus, Besut, Terengganu Malaysia; 4 Faculty of Medicine, Universiti Sultan Zainal Abidin, Kuala Terengganu, Terengganu Malaysia; 5 Department of Medical Microbiology and Parasitology, Faculty of Medicine, Universiti Teknologi MARA (UiTM), Sungai Buloh Campus, Jalan Hospital, Sungai Buloh, Selangor Malaysia; 6 Department of Pathology, Sungai Buloh Hospital, Ministry of Health Malaysia, Jalan Hospital, Sungai Buloh, Selangor Malaysia; 7 Department of Pathology, Sultanah Nur Zahirah Hospital, Ministry of Health Malaysia, Jalan Sultan Mahmud, Kuala Terengganu, Terengganu Malaysia

**Keywords:** Microbiology, Molecular biology

## Abstract

Pilus has been recently associated with pneumococcal pathogenesis in humans. The information regarding piliated isolates in Malaysia is scarce, especially in the less developed states on the east coast of Peninsular Malaysia. Therefore, we studied the characteristics of pneumococci, including the piliated isolates, in relation to antimicrobial susceptibility, serotypes, and genotypes at a major tertiary hospital on the east coast of Peninsular Malaysia. A total of 100 clinical isolates collected between September 2017 and December 2019 were subjected to serotyping, antimicrobial susceptibility test, and detection of pneumococcal virulence and pilus genes. Multilocus sequence typing (MLST) and phylogenetic analysis were performed only for piliated strains. The most frequent serotypes were 14 (17%), 6A/B (16%), 23F (12%), 19A (11%), and 19F (11%). The majority of isolates were resistant to erythromycin (42%), tetracycline (37%), and trimethoprim-sulfamethoxazole (24%). Piliated isolates occurred in a proportion of 19%; 47.3% of them were multidrug-resistant (MDR) and a majority had serotype 19F. This study showed ST236 was the most predominant sequence type (ST) among piliated isolates, which was related to PMEN clone Taiwan^19F^-14 (CC271). In the phylogenetic analysis, the piliated isolates were grouped into three major clades supported with 100% bootstrap values. Most piliated isolates belonged to internationally disseminated clones of *S. pneumoniae*, but pneumococcal conjugate vaccines (PCVs) have the potential to control them.

## Introduction

*Streptococcus pneumoniae* (pneumococcus) is a Gram-positive bacterium that frequently colonizes and becomes the normal flora of the upper human respiratory tract. Under favourable conditions, pneumococci can cause a variety of severe diseases, such as pneumonia, meningitis, and bacteremia^1,2^. Worldwide, pneumococcal infection continues to be a major cause of death and morbidity, especially among children, the elderly, and patients with comorbid diseases. According to the World Health Organization (WHO), about 808.694 children under five years of age die due to pneumonia, accounting for 15% of total death cases in 2017^3^. Malaysia is a rapidly developing country in Southeast Asia, experiencing extensive industrialization and urbanization, which is concerning from the perspective of public health as respiratory diseases, particularly pneumonia, are one of the primary causes of hospitalization and death. According to the Department of Statistics, pneumonia has been reported as the third leading cause of death in Malaysia among children under five years of age, with an estimated 3.8 per 100 000 cases per year, and among the causative agents of this disease is *S. pneumoniae*^4,5^.

The polysaccharide capsule in *S. pneumoniae* is a major virulence factor with 100 different serotypes^6^. The 7-valent pneumococcal conjugate vaccine (PCV7) was first implemented for children in the United States of America (USA) in 2000 and Europe in 2001 to reduce the burden of pneumococcal diseases. This vaccine covers serotypes 4, 6B, 9V, 14, 18C, 19F, and 23F. However, pneumococcal infection remains a severe problem due to the replacement of PCV7 serotype by non-PCV7 serotypes. 10-valent conjugate vaccine (PCV10; additional serotypes 1, 5 and 7F) was introduced in 2008 to cover a wider number of serotypes, while in early 2010, a 13-valent conjugate vaccine (PCV13; additional serotypes 3, 6A and 19A) was introduced in the USA primarily to replace PCV7 as a part of the infant immunization initiative. PCV13 was also introduced in Malaysia in 2010, with full support from paediatricians for use in childhood immunization^7^ but has yet to be widely implemented across the nation. In 2020, it was included in the Malaysia National Immunisation Programme (NIP).

Additionally, pneumococci are also equipped with a wide virulence regiment such as pneumolysin, choline-binding proteins, neuraminidase, hyaluronate lyase, autolysin, and many others. Recently, the discovery of a long filamentous, pilus**-**like structure in Gram-positive bacteria, specifically in *S. pneumoniae,* has added another factor to the pneumococcal virulence regiment. The role of pili is to enhance the ability of pneumococci to adhere to epithelial cells; at the same time, the piliated strain was found to be significantly more virulent in a murine model of invasive diseases^8^. This suggests that pneumococcal pili provide an extra advantage to initiate colonization, leading to the downstream of infection and pathogenesis process^9–11^. To date, two pili islets have been detected in pneumococci, namely, PI-1^8^ and PI-2^9^.

While a number of studies have addressed the serotype, antimicrobial resistance, and sequence type (ST) of piliated pneumococcal strains, information regarding the piliated pneumococcal isolates in the Malaysian population has been scarce. Furthermore, certain areas are still underreported, particularly on the east coast of Peninsular Malaysia, where the process of urbanization is less extensive than that on the west coast. The emergence of a subpopulation carrying the pilus trait which is not common in Gram-positive bacteria should not be underestimated. Therefore, this study was undertaken to determine the serotype distribution, antimicrobial resistance, virulence gene profile, occurrence of pilus genes, and the genotypic characteristics of pneumococci and piliated isolates at a major tertiary hospital on the east coast of Peninsular Malaysia.

## Results

### Demographic data

A total of 100 *S. pneumoniae* isolates were collected over a period of 28 months, between September 2017 and December 2019. Of these, the most frequent site of isolation was sputum (*n* = 40; 40%) followed by blood (*n* = 38; 38%), eye (*n* = 15; 15%), pus (*n* = 4; 4%), bronchial aspirate (*n* = 2; 2%), and swab (*n* = 1; 1%). The information on disease and admission status of patients was restricted and the scope of analysis was based on the patient’s demographics, isolation sites, and phenotypic and genotypic characteristics of the isolates only. Isolates from sterile sites such as blood were categorized as from invasive sites (*n* = 38; 38%), and the rest of non-sterile areas as non-invasive sites (*n* = 62; 62%). Demographic analysis showed the isolation frequency of pneumococcal isolates was slightly higher in male (*n* = 54; 54%) than in female subjects (*n* = 46; 46%). Distribution of pneumococcal isolates according to age groups was as follows: ≤ 5 years; *n* = 24, 24%, 5 >  ≤ 12 years; *n* = 5, 5% 12 >  ≤ 50 years; *n* = 35, 35% and 50 > years; *n* = 36, 36% (Table 1).

**Table 1:** Serotype distribution of pneumococcal isolates in relation to patient age group.

Serotype	≤ 5 years (*n* = 24)	5 > ≤ 12 years (*n* = 5)	12 > ≤ 50 years (*n* = 35)	50 > years (*n* = 36)	Total (*n* = 100)
1	1	–	–	3	4
6A/B	6	1	7	2	16
7A/F	1	–	–	–	1
7C	–	–	–	1	1
8	–	–	–	1	1
11A/D	–	–	1	1	2
12F	–	–	–	1	1
14	5	1	5	6	17
15B/C	1	1	2	3	7
18A/B/C	1	1	–	1	3
19A	3	–	4	4	11
19F	3	–	5	3	11
20	–	–	2	2	4
23A	–	–	–	1	1
23B	–	–	–	1	1
23F	1	–	7	4	12
Non-typeable	2	1	2	2	7

### Antimicrobial susceptibility profile

**Figure 1:**
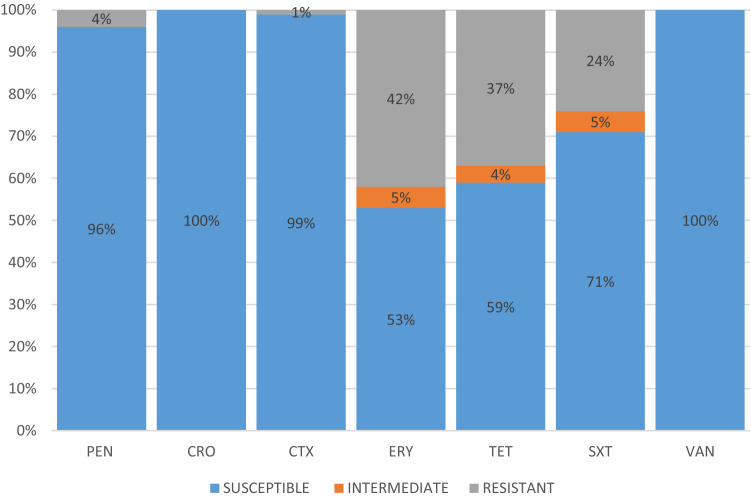
Antimicrobial susceptibility pattern among the collection of 100 isolates. Susceptibility test by E-test (MIC determination) for penicillin (PEN), ceftriaxone (CRO) and cefotaxime (CTX). Susceptibility test by disk diffusion for tetracycline (TET), erythromycin (ERY), trimethoprim-sulfamethoxazole (SXT) and vancomycin (VAN).

Among beta lactam antibiotics, non-meningitis susceptibility breakpoint was used, whereby penicillin resistance was observed in four (4%) isolates and cefotaxime resistance in another isolate; all were from blood. Since data on the disease status of the patients were not accessible, only isolate from cerebrospinal fluid (CSF) was associated with meningitis, while isolates from other isolation sites were assumed as non-meningitis, but none of the isolates in this study were from CSF. For the other antibiotics, the majority of isolates were resistant to erythromycin (*n* = 42; 42%), tetracycline (*n* = 37; 37%), and trimethoprim-sulfamethoxazole (*n* = 24; 24%), with 18% (18/100) of the total collection being MDR. All isolates were susceptible to vancomycin. The distribution of antimicrobial susceptibilities for all isolates in this study is shown in Fig. 1.

### Distribution of serotypes

Seventeen different serotypes with six different serogroups were determined. The most prevalent was serotype 14 (*n* = 17; 17%), followed by serotypes 6A/B (*n* = 16; 16%), 23F (*n* = 12; 12%), 19A (*n* = 11; 11%), 19F (*n* = 11; 11%), 15B/C (*n* = 7; 7%), 1 (*n* = 4; 4%), 20 (*n* = 4; 4%), 18A/B/C (*n* = 3; 3%), 11A/D (*n* = 2; 2%), 7A/F (*n* = 1; 1%), 12F (*n* = 1;%), 23A (*n* = 1;1%), 8 (*n* = 1; 1%), 23B (*n* = 1; 1%), and 7C (*n* = 1; 1%). Seven isolates were designated as non-typeable (NT) as they were not amplified for any of the molecular targets. Most of them were from non-invasive site, while five isolates (71.4%) were from sputum, one isolate was from eye (14.3%), and one (14.3%) from blood. Overall, serotypes 14, 6A/B, 19F, 23F, and 19A accounted for more than half of the total isolates (*n* = 67; 67%) belonging to PVC13.

Serotype distributions varied according to patients’ age group (Table 1). Serotype 6A/B (*n* = 6; 25%) showed a high percentage, followed by serotype 14 with 5 isolates (20.8%) in subjects of ≤ 5 years. Among subjects in the 12 >  ≤ 50 year-old group, the most common serotypes were 23F (*n* = 7; 20%), 6A/B (*n* = 7; 20%), 19F (*n* = 5; 14.3%), 14 (*n* = 5; 14.3%), and 19A (*n* = 4; 11.4%). Serotype 14 (*n* = 6; 16.7%) was the predominant serotype for > 50 year-old age groups. The non-PCV13 vaccine type had a frequency of occurrence of 25% (25/100), whereby serotype 15B/C (*n* = 7; 7%) was the most prevalent, followed by serotypes 20 (*n* = 4; 4%), 11A/D (*n* = 2; 2%), and many others. Overall immunization coverage frequency of isolates targeted by PCV10 and PCV13 was 64% (64/100) and 75% (75/100), respectively.

Isolates of serotype 19F (*n* = 11) showed frequent resistance to tetracycline (*n* = 10/11) and trimethoprim-sulfamethoxazole (*n* = 6/11). Serotypes 6A/B (*n* = 16) had a higher frequency of penicillin resistance (*n* = 3/16), while serotype 14 (*n* = 17) showed a high frequency of resistance to erythromycin (*n* = 9/17). Meanwhile, MDR was found in 18.4% of the pneumococcal isolates from the invasive site (*n* = 7/38). Nevertheless, in the overall analysis, taking the isolation site as the baseline, there was no significant association in relation to gender, age, vaccine serotype, multidrug-resistance, and pilus genes (Table 2).

**Table 2:** Isolation sites of the pneumococcal isolates in relation to gender, vaccine serotypes, and multidrug-resistance.

Site of isolation	Age		Gender		Pneumococcal vaccine (PCV)		Antibiotic pattern		Pilus genes	
	≤ 12 years (*n* = 28)	12 > years (*n* = 72)	Male (*n* = 57)	Female (*n* = 43)	Vaccine serotype (*n* = 75)	Non-vaccine serotype (*n* = 25)	^c^MDR (*n* = 20)	^d^Non-MDR (*n* = 80)	Piliated strains (*n* = 19)	Non-piliated strains (*n* = 71)
^a^Invasive (n = 38)	13	25	24	14	30	8	8	30	6	32
^b^Non-invasive (n = 62)	15	47	33	29	45	17	12	50	13	49
*p*-value	0.279		0.330		0.475		0.539		0.4105	

^a^Invasive site (blood).

^b^Non-invasive site (sputum, eye, pus, bronchiol, swab).

^c^MDR = resistance to ≥ 3 antibiotics.

^d^Non-MDR = resistance to < 3 antibiotic(s).

### Occurrence of virulence and pilus genes

All the tested virulence genes, namely, *ply, lytA*, *cpbA, pavA*, and *pspA*, were detected by PCR in all 100 pneumococcal isolates. However, only 19 isolates (19%) possessed at least one of the pilus genes. Among these, 13 isolates (13%) were detected for PI-1 alone, one isolate (1%) for PI-2 alone, and five isolates (5%) presented both genes (PI-1 + PI-2). Pili were detected in isolates from years 2017 (*n* = 7), 2018 (*n* = 4), and 2019 (*n* = 8). In terms of distribution, the isolation sites among the piliated isolates were from blood at 31.6% (6/19), while non-invasive sites were from eye (*n* = 5/19; 26.3%), sputum (*n* = 5/19; 26.3%), pus (*n* = 1/19; 5.3%), bronchial aspirate (*n* = 1/19; 5.3%), and swab (*n* = 1/19; 5.3%).

In relation to antibiotic susceptibility pattern, isolates with PI-1 alone and both PI-1 + PI-2 were frequently resistant to erythromycin (*n* = 14; 73.7%), tetracycline (*n* = 12; 63.1%), and trimethoprim-sulfamethoxazole (*n* = 10; 52.6%). The only PI-2 alone-isolate was susceptible to all antibiotics. Of these PI-1 alone and both PI-1 + PI-2 isolates, nine (47.3%) were MDR. The majority of PI-1 alone and both PI-1 + PI-2 isolates belonged to serotype 19F (*n* = 8; 42.1%), followed by serotype 6A/B (*n* = 4; 21.1%), 19A (*n* = 3; 15.8%), and 14 (*n* = 2; 10.5%). PCV13 covered a vast majority of the isolates with PI-1 alone and both PI-1 + PI-2. The one PI-2 alone isolate showed serotype 20, which was a non-vaccine serotype. The frequency of piliated pneumococcal isolates targeted by PCV10 and PCV13 was 73.7% (*n* = 14) and 89.5% (*n* = 17), respectively.

### Multilocus sequence typing (MLST) analysis

MLST was conducted only on isolates that possessed pilus genes (*n* = 19). All the respective amplicons from the isolates matching the expected DNA band size were successfully sequenced, yielding 11 distinct STs and seven different clonal complexes (CC) (Table 3). The predominant one was ST236 (*n* = 8; 42.1%), followed by ST671 (*n* = 2; 10.5%), ST271 (*n* = 1; 5.3%), ST90 (*n* = 1; 5.3%), ST700 (*n* = 1; 5.3%), ST11811 (*n* = 1; 5.3%), ST320 (*n* = 1; 5.3%), ST386 (*n* = 1; 5.3%), ST62 (*n* = 1; 5.3%), ST2648 (*n* = 1; 5.3%), and a new novel sequence type, ST15604. The novel ST15604 isolate originated from the sputum of a 7-year-old child, was susceptible to all antibiotics, and had serotype 8 carrying PI-1 gene.

**Table 3:** Genotypic characteristics of the piliated pneumococcal isolates in relation to serotypes and antibiotic susceptibilities.

ST	CC	Serotype	PMEN clones	Number of isolates resistant to antibiotics							MDR (*n*)	PI-1 alone	PI-2 alone	PI-1 + PI-II
				PEN^a^	CRO^a^	CTX^a^	TET^b^	ERY^b^	SXT^b^	VAN^b^				
ST236	CC271	19F (*n* = 6)	Taiwan^19F^-14	S (6)	S (6)	S (6)	R (6)	R (6)	S (2), I (1), R (3)	S (6)	Yes (3), No (3)	4	–	2
		19A (*n* = 1)		S (1)	S (1)	S (1)	S (1)	S (1)	S (1)	S (1)	No	–	–	1
		6A/B (*n* = 1)		S (1)	S (1)	S (1)	R (1)	R (1)	R (1)	S (1)	Yes	–	–	1
ST271		19F (*n* = 1)	SLV of Taiwan^19F^-14	S (1)	S (1)	S (1)	R (1)	R (1)	R (1)	S (1)	Yes	–	–	1
ST320		19A (*n* = 1)	DLV of Taiwan^19F^-14	R (1)	R (1)	R (1)	R (1)	R (1)	R (1)	S (1)	Yes	1	–	
ST2648		19F (*n* = 1)	DLV of Taiwan^19F^-14	R (1)	R (1)	R (1)	R (1)	R (1)	R (1)	S (1)	Yes	1	–	
ST671	CC156	14 (*n* = 2)	TLV of Spain^9V^-3	S (1), R (1)	S (2)	S (2)	S (2)	R (2)	S (1), R (1)	S (2)	No (2)	2	–	–
ST15604		8 (*n* = 1)	SLV Spain^9V^-3	S (1)	S (1)	S (1)	S (1)	S (1)	R (1)	S (1)	No	1	–	
ST90	CC90	6A/B (*n* = 1)	Spain^6B^-2	S (1)	S (1)	S (1)	R (1)	R (1)	R (1)	S (1)	Yes	1	–	
ST11811	CC146	6A/B (*n* = 1)	TLV Spain^9V^-3	S (1)	S (1)	S (1)	S (1)	S (1)	R (1)	S (1)	No	1	–	
ST386	CC386	6A/B (*n* = 1)	DLV Poland^6B^-20	S (1)	S (1)	S (1)	R (1)	R (1)	R (1)	S (1)	Yes	1	–	
ST62	CC53	20 (*n* = 1)	DLV of Netherlands^8^-33	S (1)	S (1)	S (1)	S (1)	S (1)	S (1)	S (1)	No	–	1	
ST700	CC230	19A (*n* = 1)	SLV Denmark^14^-32	S (1)	S (1)	S (1)	S (1)	S (1)	S (1)	S (1)	No	1	–	

*ST* sequence type, *CC* clonal complex, *PMEN* Pneumococcal Molecular Epidemiology Network, *SLV* single-locus variant, *DLV* double-locus variant, *TLV* triple-locus variant, *PEN* penicillin, *TET* tetracycline, *ERY* erythromycin, *CRO* ceftriaxone, *CTX* cefotaxime, *SXT* trimethoprim-sulfamethoxazole, *VAN* vancomycin., *MDR* multidrug resistance;

^a^Susceptibility test by E-test (MIC determination) for penicillin, ceftriaxone and cefotaxime.

^b^Susceptibility test by disk diffusion for tetracycline, erythromycin, trimethoprim-sulfamethoxazole and vancomycin.

Nineteen piliated isolates presented STs similar to 11 of the 43 clones recognized by the Pneumococcal Molecular Epidemiology Network (PMEN), sharing at least five MLST alleles with those PMEN clones. Nine isolates had the same ST as Taiwan ^19F^-14 and Spain^6B^-2, three strains were single-locus locus variant (SLV) of Taiwan^19F^-14, Denmark^14^-32, and Spain^9V^-3, four isolates were double-locus variant (DLV) of Taiwan^19F^-14, Poland^6B^-20, and Netherlands^8^-33**,** and three isolates were triple-locus variant (TLV) of Spain^9V^-3 and Spain^6B^-2.

### Phylogenetic analysis

**Figure 2:**
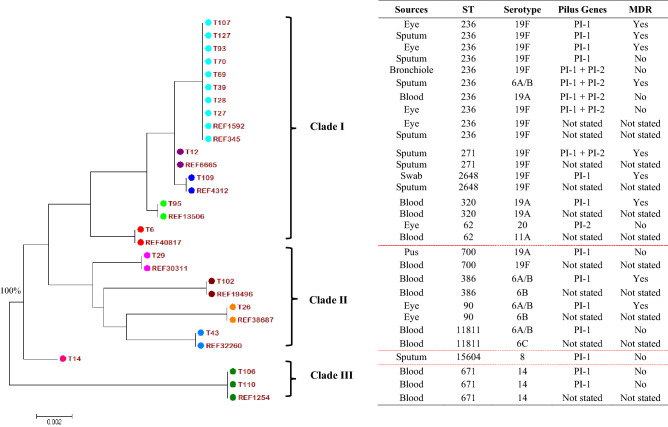
Phylogenetic analysis of the piliated pneumococcal isolates. Phylogenetic analysis revealed three clades among piliated pneumococcal isolates with clade I being predominant and exhibiting mostly serotype 19F of ST236, and all the PI-1 alone and both PI-1+PI-2 isolates. Bootstrap sampling at 1000 replicates showed 100% at all branching. The distribution of sources, sequence type (ST), serotype, pilus genes and multidrug resistance for respective isolates are shown in the right columns. The reference sequences were retrieved from the MLST database comprising identity (ID) numbers 1592 (Taiwan), 345 (Taiwan), 6665 (South Korea), 4312 (Vietnam), 13506 (Malaysia), 40817 (Japan), 30311 (Malawi), 19496 (India), 38687 (China), 32260 (United States) and 1254 (United States); ID number was preceded by REF indicating reference sequence, and ID labelled with T (Terengganu) and numbers represent the isolates in this study. T14 is a newly assigned ST (REF sequence is not available).

All nucleotide sequences of the seven housekeeping genes for the respective isolates were aligned in the specified order^12^ and subjected to phylogenetic analysis among all the 19 piliated isolates (labelled with T followed by numbers) and 11 reference sequences from the MLST database (labelled with REF followed by identity number). The tree was basally rooted to strain numbers T106 and T110, assigned as clade III and branched out into two bigger clades I and II with 100% bootstrap confidence interval values at all branching (Fig. 2). Among the three clades, there was one major group consisting of 12 isolates from this current study (Clade I), while others were small clades comprising two and four isolates (clade II and clade III). The clades also showed clear segregation among the STs, where clade I represented predominantly ST236, followed by ST271, ST2648, ST320, and ST62; clade II for ST700, ST386, ST90, and ST11811; clade III for ST671 for both isolates; and a single ST15604 (novel) in its own lineage. With the exception of the novel ST, all reference sequences of the different STs were clustered according to their respective similar ST of the isolates in this study.

## Discussion

Molecular studies involving pneumococcal isolates from the east coast states of Peninsular Malaysia are scarce. The current study is the first report to address piliated pneumococci in this eastern region. Based on the 2018 Malaysia economic report^13^, Terengganu has a lower gross domestic product (GDP) than some states on the west coast, but it is famous as a local and international tourist destination owing to its beautiful beach. This could affect the dynamics of pneumococcal dissemination, which is why the genetic background of pneumococcal isolates from this area is of interest. In accordance with a previous study^14^, our finding also showed sputum (40%) and blood (38%) as the most common sources with certain serotype distribution pattern. Meanwhile, a slight male predominance was noted in the study population. Nonetheless, statistical analysis showed that the isolation sites were not significantly correlated with gender, age, vaccine serotype, multidrug-resistance, and pilus genes. Subsequently, the analysis addressed the molecular aspects, particularly the piliated elements.

Serotype 14 was the most predominant serotype among pneumococcal isolates in our collection. A previous study showed serotype 19F as the common serotype among isolates from different sites of isolation from various Malaysian hospitals^15^. On the other hand, the frequency of occurrence of serotype 19F was lower in this current study; it is not known whether this is the serotype pattern in the east or potentially a natural serotype shift despite the lack of pneumococcal immunization. The PCV has only been made available privately and is probably more broadly available and affordable in some developed states in the west. However, since 2020, it has been part of the national immunization program. Meanwhile, the current study showed that serotype 15B/C predominated among non-vaccine serotypes. A previous study reported that serotype 15B/C emerged as the predominant non-vaccine serotype and was widely disseminated^16^. Additionally, serotype predominance in relation to age group was observed in a previous study that revealed serotype 14 as the most common serotype in children less than 48 months of age in Russia^17^. This was in agreement with our finding that serotype 14 and 6A/B was predominant among ≤ 5 year-old group. Serotype 14 was also found to be the most prevalent serotype among invasive paediatric isolates in a recent study in Malaysia^7^.

The incidence of antibiotic resistant pneumococci has been increasing in Asia^18,19^. However, a lower resistance frequency was observed for penicillin (4%) in this current study, consistent with a previous report from Malaysia that also demonstrated a low prevalence of penicillin resistance at only 5.6%^20^. On the other hand, a higher prevalent frequency (26.8%) of penicillin resistance was reported in Korea^21^. Meanwhile, among the 100 isolates in this study, a high incidence of resistance was observed against erythromycin (42%), tetracycline (37%), and trimethoprim-sulfamethoxazole (24%). A similar trend was documented by a recent study in Malaysia, with resistance to both erythromycin and tetracycline being 42.9%^7^. Co-resistance against these antibiotics was the common MDR pattern among the isolates, which was also found in other Asian countries^22,23^. Nevertheless, the isolates remained largely susceptible to ceftriaxone, cefotaxime, and vancomycin, which were consistently reported in a previous study in Malaysia as well^7^.

The pneumococcal regiments of virulence genes tested in this study are commonly present in pneumococci worldwide, as also observed in this study with the exception of pili^24^. Pilus is a sex organ, but it has been suggested that pili additionally play a significant role in pathogenesis by promoting adhesion and colonization of host tissue, as well as cellular invasion to provide an extra advantage to the isolates^9^. In previous studies, PI-1 was found to occur in 16.6% -35%^25,26^ and PI-2 in approximately 9.5–20% of pneumococcal isolates^27,28^. The prevalence of piliated isolates in this study was within the range, with only 19% of isolates possessing at least one of the pilus genes. In similar fashion, a previous local Malaysian study revealed 20.6% of isolates amplified for PI-1 and 14.0% for PI-2^29^. Interestingly, despite their lower occurrence, 6 out of 19 of the piliated isolates in this current study were collected from blood (31.6%). Likewise, a Canadian study reported that blood was the common sources for piliated isolates^30^. In addition, about 47.3% of piliated isolates in our study were also MDR, being resistant to erythromycin, tetracycline, and trimethoprim-sulfamethoxazole. Earlier evidence showed that MDR was strongly associated with piliated isolates^31^.

While serotype 14 was the most frequent in our study, serotype 19F was predominant, being associated with the presence of PI-1 alone and both PI-1 + PI-2. Similarly, a study in China found that serotype 19F was the most frequently associated with PI-1^32^. Overall, the majority of piliated isolates in our study belonged to PCV13 exhibiting serotypes 19F, 19A, 6A/B, and 14. This is in agreement with previous reports regarding a correlation between piliated pneumococci isolates and vaccine-serotypes, commonly 19F, 19A, 14, and 6A/6B, which are included in PCV13^25,33^. On the other hand, Regev-Yochay and colleagues reported the emergence of PI-1 piliated isolates of the non-vaccine type after the implementation of PCV7 in the USA^34^. This served to highlight the implications of PCV for the advent of a new clonal expansion, which could be possibly facilitated by the pili properties as well, especially in establishing successful colonization.

Although we characterized only 19 piliated pneumococcal isolates for MLST, we revealed 11 different STs, including one newly assigned ST in the present study. From the MLST perspective, PI-1 and PI-2 are frequently found in pneumococcal lineages that are related to international PMEN clones. ST236 was the most prevalent among piliated isolates in our collection and belonged to CC271. ST236 is also the dominant lineage in Southeast Asia and many parts of the world, but there are limited studies discussing the association of ST236 with the appearance of pilus genes. In our study, the majority of PI-1 alone and both PI-1 + PI-2 isolates associated with ST236 were related to PMEN clone Taiwan^19F^-14 (CC271), at the same time being vaccine type (19F and 19A) with MDR phenotype. This is in concordance with a previous study in Japan that reported the incidence of ST236 associated with PI-1 isolates and the widely distributed multidrug-resistant serotype 19F clone Taiwan^19F^-14^35^. Meanwhile, MLST analysis in our study showed that ST271 and ST320 of CC271, harbouring PI-1 alone and both PI-1 + PI-2 genes, were also associated with MDR clone Taiwan^19F^-14. CC271 has been associated with the multidrug-resistant pneumococcal clone (Taiwan^19F^-14), which has been described worldwide as carrying a piliated element^32^.

The molecular analysis of *S. pneumoniae* in this study revealed several genetic lineages, namely, ST90, ST386, and ST671, which were associated with PI-1 alone and the majority of them from the invasive site carrying the MDR phenotype; those STs were also related to the widely disseminating PMEN clones, including Spain ^6B^-2 (CC90), Poland^6B^-20 (CC386), and Spain^9V^-3 (CC156), respectively. A previous study reported that PI-1 was primarily associated with PMEN clones, such as Spain^6B^-2 (CC156) and Taiwan^19F^-14 (CC320), with all of them being vaccine-serotype covered by PCV13^31^. In our collection, the only PI-2 isolate was found in ST62 and associated with Netherland^8^-33 (CC53) clone. Zahner and colleagues also reported that PI-2 isolates from the invasive site in their US study were linked to Netherland^8^-33 clone (ST62) but had serotype 11A, which is a non-vaccine type^28^. This indicated that the presence of pili was attributed to homogenous clonal expansion and the majority of the MDR clones were occupied by those piliated strains.

The phylogenetic tree indicated a close genetic lineage among several piliated isolates, especially those carrying both pilus genes. The most dominant clade I consisted of isolates that exhibited mainly ST236, ST271, ST320, and ST2648 of CC271 carrying PI-1 alone and both PI-1 + PI-2. Most isolates from this clade showed a similar MDR pattern and vaccine-serotype as well. There were a few strains in the dominant clade I manifesting different serotypes, which could potentially be due to capsular switches of serotype^36^. A notable observation was two piliated isolates related to ST236 expressing serotypes 19A and 6A/B. The only PI-2 isolate in clade I had serotype 20, which was a non-vaccine type. Clade II consisting of PI-I alone isolates was the second major clade that represented the diversity of STs, including ST700, ST386, ST90, and ST11811. Most of the piliated strains from this clade were of serotype 6A/B, and only one isolate had serotype 19A. ST386 and ST90 isolates had the MDR phenotype. The emergence and expansion of the MDR serotype 6C-CC386 lineage in Brazil was reported after universal use of PCV^37^, while our study revealed serotype 6A/B for ST386, suggesting a potential capsular switch event. Such an observation suggested that pili might help this lineage to emerge after the selective pressure of the vaccination.

Meanwhile, clade III consisted of two ST671 isolates that had PI-I alone. They had similar serotype 14 and non-MDR and they were both from the invasive site (blood), which reflected their close association. The new ST15604 belonged to its own lineage in the phylogenetic tree, having PI-1 alone and serotype 8, which was a non-vaccine type. ST15604 could possibly emerge differently from others as it was located more basally and distinct in the tree structure. All in all, the analysis showed that the majority of the piliated pneumococcal isolates in this study displayed close genetic correlation due to being clustered together, with some exhibiting similar serotypes and STs. It can thus be deduced that they could have initially derived from a common origin and subsequently disseminated within the population.

Nevertheless, the phylogenetic analysis relies on mutation as per the MLST principle. Gene *ddl* that is used in the *S. pneumoniae* MLST scheme had been previously linked to sequence replacement due to the ‘hitch hiking’ effect. The reason for this is that it is located near the penicillin-binding protein 2b gene (*pbp2b*) that is well-known with its mosaic structure due to sequence recombinational replacement leading to penicillin resistance^38^. Such a mutational event may also affect the nearby *ddl* gene to end up with similar sequence diversity. Our study did not take into consideration such potential sequence replacement in *ddl* gene that could affect the phylogenetic output due to supporting data limitations. In addition, because of research scarcity, the frequency of such *ddl* gene-diversity-related event is unknown. Further analysis utilizing whole genome sequencing is highly warranted to elucidate this matter and to provide a better understanding of the genetic organization and evolution of the piliated isolates.

## Conclusion

This study carried out a phenotypic and genotypic analysis of piliated pneumococcal isolates at a major tertiary hospital on the east coast of Peninsular Malaysia. This tourist attraction area was chosen as studies on the topic in question were scarce there. We found that the pneumococcal pilus islet was associated with clonal spread involving many serotypes such as 19F, 19A, and 6A/B, which are mostly covered by PCVs. The predominant ST of the piliated isolates was ST236. This was linked to the clone Taiwan^19^-14. ST236, ST271, ST320, ST90, ST386, and ST2648 of piliated isolates, which have also been associated with a high frequency of MDR. Based on these findings, it can be suggested that global pneumococcal lineages have been disseminated in this area and pili could play a role in the spread of antibiotic resistant clones, as supported by the phylogenetic analysis as well. Fortunately, the vaccine-serotypes exhibited by these clones can be controlled by PCVs, but a potential serotype switch may rule out isolates from the vaccine coverage. This warrants continuous monitoring, particularly when the PCV has been fully implemented.

## Materials and methods

### Bacterial isolates

Clinical *S. pneumoniae* isolates were collected from the Microbiology Laboratory, Department of Pathology, Hospital Sultanah Nur Zahirah (HSNZ), Kuala Terengganu, Terengganu between September 2017 and December 2019. This is a tertiary hospital with more than 800 beds  serving the capital city of the state of Terengganu and nearby areas on the east coast of Peninsular Malaysia. Each clinical isolate was obtained from a different individual from invasive (blood) and non-invasive sites (sputum, eye, pus, bronchial aspirate and swab of a non-sterile area). The ages of the patients ranged from one month to 82 years old. The information on disease and admission status of patients was not accessible and therefore was excluded from analysis. *S. pneumoniae* was confirmed by bile solubility and susceptibility to ethylhydrocupreine disc (optochin). *S. pneumoniae* ATCC 49,619 was included as a reference strain in all analyses.

### Antimicrobial susceptibility test

The antimicrobial susceptibility of pneumococcal isolates was assessed by the disk diffusion method (Oxoid, USA) for erythromycin, tetracycline, trimethoprim-sulfamethoxazole, and vancomycin. Meanwhile, the minimal inhibitory concentrations (MIC) of penicillin, ceftriaxone, and cefotaxime were determined using the E-test method (BioMérieux, France). Both methods followed the CLSI procedures and interpretation guidelines, taking into consideration the different MIC criteria for isolates from meningitis and non-meningitis^39^. Both assays were tested on Mueller Hinton Agar (Isolab) with 5% sheep blood, incubated at 37 °C in 5% CO_2_. Isolates resistant to three or more antimicrobial agents were defined as multidrug-resistant (MDR)^40^.

### Genomic DNA extraction

Overnight pure culture on Columbia agar with 5% sheep blood was used for DNA extraction via the GeneAll Exgene kit (GeneAll Biotechnology Co.Ltd, Korea) as per the manufacturer’s instructions.

### Detection of *S. pneumoniae* virulence and pilus genes

*S. pneumoniae* virulence (*ply, lytA, cbpA, pavA* and *pspA*) and pilus genes (*rlrA, rrgA, rrgC* and *sipA*) were amplified using PCR assays with primers and running conditions, as previously described (see Supplementary Table S1)^10,28,41–46^. All PCR products were electrophoresed on 1.7% agarose gel with Etb “Out” Nucleic Acid Staining (Yeastern Bio) for 1 h at 80 V.

### Determination of capsular types

Pneumococcal capsular types were deduced by multiplex PCR using published primers recommended by the Center for Disease Control and Prevention (CDC)^47^. Primers were divided into six multiplex sets named A, B, C, D, E, and F, as previously described^29,48^. The *cpsA* gene found in all known pneumococcal serotypes was used as the positive control, while the 100 bp plus DNA ladder marker (GeneDirex) was used for molecular weight reference.

### Multilocus sequence typing (MLST)

Only pili-carrying pneumococcal isolates were subjected to MLST. The internal fragments of seven housekeeping genes, namely, *aroE, gdh, gki, recP, spi, xpt*, and *ddl*, were amplified by PCR, as previously described^12^. Sequences were submitted to the MLST database (http://spneumoniae.mlst.net) for the assignment of allelic profiles and sequence type (ST). New alleles and ST were submitted to the curator of the MLST website for verification. The PHYLOViZ software was used for assigning the isolates for clonal complexes (CC) defined as cluster sharing at least five out of seven alleles. ST profiles were inferred to Pneumococcal Molecular Epidemiology Network (PMEN) clones in the PMEN database (http://www.sph.emory.edu/PMEN/pmen_table1.html) to identify the close lineage of circulating clones.

MLST phylogenetic analysis based on the seven housekeeping genes^12^ was determined by using Molecular Evolutionary Genetics Analysis version 7 (MEGA7). Appropriate reference sequences ID 1592, 345, 6665, 4312, 13506, 40817, 30311, 19496, 38687, 32260, and 1254 for respective STs were retrieved from the MLST database and included in the analysis as control. The phylogenetic tree was constructed by MEGA7 using the maximum-likelihood method based on the Tamura-Nei model, while the reliability of the tree was estimated via bootstrap analysis with 1000 replicates.

### Statistical analysis

Chi-square was used to compare the demographic characteristics of the patients with phenotypic and genotypic variables of the pneumococcal isolates. Statistical significance was indicated by *p* < 0.05.

### Ethical considerations

Ethical approval for this study was granted by the Medical Research and Ethics Committee of the Malaysian Ministry of Health, National Medical Research Register (approval no. NMRR 17-1025-35696). The study used de-identified pneumococcal isolates collected at the microbiology laboratory of the hospital. Since it did not meet the definition of research involving human subjects, informed consent was not required for this study; only data related to isolation site, age, and gender of the patients associated with the isolates were provided and were not traceable to the sampled individuals. Additionally, all methods were carried out in accordance with the relevant guidelines and regulations.

## Supplementary Information

Supplementary Information
