# Supplementary material for: Antimicrobial susceptibility, serotype distribution, virulence profile and molecular typing of piliated clinical isolates of pneumococci from east coast, Peninsular Malaysia
Source: Sci Rep. 2021 Apr 15;11:8220. doi: 10.1038/s41598-021-87428-z (PMC8050075; doi:10.1038/s41598-021-87428-z)
Supplement: Supplementary file 1 — Supplementary Information [file 41598_2021_87428_MOESM1_ESM.docx]

**Antimicrobial susceptibility, serotype distribution, virulence profile and molecular typing of piliated clinical isolates of pneumococci from east coast, Peninsular Malaysia**

Nurul Diana Dzaraly^1^, Mohd Nasir Mohd Desa^1^*, AbdulRahman Muthanna^1^, Siti Norbaya Masri^2^, Niazlin Mohd Taib^2^, Zarizal Suhaili^1,3^, Nurshahira Sulaiman^1^, Nurul Hana Zainal Baharin^1^, Cheah Yun Shuan^1^, Zarina Ariffin^1^, Nor Iza A. Rahman^4^_,_ Farahiyah Mohd Rani^4^, Navindra Kumari Palanisamy^5^, Tuan Suhaila Tuan Soh^6^, Fatimah Haslina Abdullah^7^.

^1^Department of Biomedical Sciences, Faculty of Medicine and Health Sciences, Universiti Putra Malaysia, UPM Serdang, Selangor

^2^Department of Medical Microbiology, Faculty of Medicine and Health Sciences, Universiti Putra Malaysia, UPM Serdang, Selangor

^3^School of Animal Science, Aquatic Science and Environment, Faculty of Bioresources and Food Industry, Universiti Sultan Zainal Abidin, Besut Campus, Besut, Terengganu

^4^Faculty of Medicine, Universiti Sultan Zainal Abidin, Kuala Terengganu, Terengganu

^5^Department of Medical Microbiology and Parasitology, Faculty of Medicine, Universiti Teknologi MARA (UiTM), Sungai Buloh Campus, Jalan Hospital, Sungai Buloh, Selangor

^6^Department of Pathology, Sungai Buloh Hospital, Ministry of Health Malaysia, Jalan Hospital, Sungai Buloh, Selangor

^7^Department of Pathology, Sultanah Nur Zahirah Hospital, Ministry of Health Malaysia, Jalan Sultan Mahmud, Kuala Terengganu, Terengganu

*** Corresponding author:**

[mnasir@upm.edu.my](mailto:mnasir@upm.edu.my)

| **Gene** | **Forward primer**  **sequence (5’ to 3’)** | **Reverse primer**  **sequence (5’ to 3’)** | **Amplicon**  **size (bp)** | **Annealing**  **temperature**  **(◦C)** | **References** |
| --- | --- | --- | --- | --- | --- |
| *rrgA* | CTCTAGGAGGGATCTTCTTTATCATC | CTACAGCCGTTGTTCGATTGTCC | 140 | 55 | ^10^ |
| *sipA* | CTCTAGGAGGGATCTTCTTTATCATC | CTACAGCCGTTGTTCGATTGTCC | 550 | 55 | ^28^ |
| *rlrA* | TCTGATAGATGAGACGCTGTTG | CTCCGCTTCTTTCTACTACAAG | 1177 | 55 | ^41^ |
| *ply* | ATTTCTGTAACAGCTACCAACGA | GAATTCCCTGTCTTTTCAAAGTC | 348 | 53 | ^42^ |
| *lytA* | CAGCGGTTGAACTGATTGA | TGGTTGGTTATTCGTGCAA | 173 | 53 | ^43^ |
| *pspA* | CCGGATCCAGCGTCGCTATCTTAGGGGCTGGTT | CCACATACCGTTTTCTTGTTTCCAGCC | 800–1500 | 55 | ^44^ |
| *rrgC* | GCTCTGTGTTTTTCTCTTGTATGG | ATCAATCCGTGGTCGCTTGTTATTTTTA | 1030 | 55 | ^45^ |
| *cbpA* | AGTTGATTGGCTTGACCTTG | CTACACTAGCTACTCC | 300 | 53 | ^46^ |
| *pavA* | TGAACTGATTCGTCGTGTTG | TCAGCGATTTCTTCCAGTCC | 381 | 53 | ^46^ |

**Supplementary Table S1. Details of primers used in detecting virulence and pilus genes**
